# Magnetic Resonance Imaging Features of Pediatric Coxsackievirus Encephalitis

**DOI:** 10.5334/jbsr.1685

**Published:** 2019-01-09

**Authors:** Yang-Kai Fan, Yu-Peng Liu

**Affiliations:** 1 Hsinchu Mackay Memorial Hospital, TW

**Keywords:** coxsackievirus, encephalitis, enterovirus, magnetic resonance imaging, pediatric

## Abstract

Coxsackievirus, a common pathogen causing pediatric infection, typically causes a mild, nonspecific illness with low-grade fever, but can cause severe illness on rare occasions. Encephalitis is an uncommon but important complication of coxsackievirus infection. The magnetic resonance imaging (MRI) findings of coxsackievirus B3 (CVB3) encephalitis have not been reported in the literature, and there are few descriptions of MRI findings of encephalitis induced by coxsackieviruses of other serotypes. We report the MRI findings of CVB3 encephalitis in a two-year-old girl, review the MRI findings in previously reported cases of pediatric coxsackievirus encephalitis, and present the MRI characteristics of coxsackievirus encephalitis.

## Introduction

Coxsackievirus is a single-stranded RNA virus and belongs to the family Picornaviridae and the genus Enterovirus, which also includes poliovirus and echovirus. Coxsackieviruses are divided into group A and group B viruses. At least 23 serotypes of group A viruses and six serotypes of group B viruses are recognized. Most coxsackievirus infections are asymptomatic or result in only mild illness, such as nonspecific febrile illness, rashes, or mild upper respiratory tract infections, but they can also cause serious diseases such as pericarditis, myocarditis, pancreatitis, or encephalitis. Because encephalitis is a rare presentation of coxsackievirus infection, there is very limited information on the MRI findings of coxsackievirus encephalitis in the literature. We present a case of CVB3 encephalitis in a two-year-old girl and compare the MRI findings to previously reported cases of coxsackievirus encephalitis and other types of enteroviral encephalitis.

## Case report

**Figure 1:**
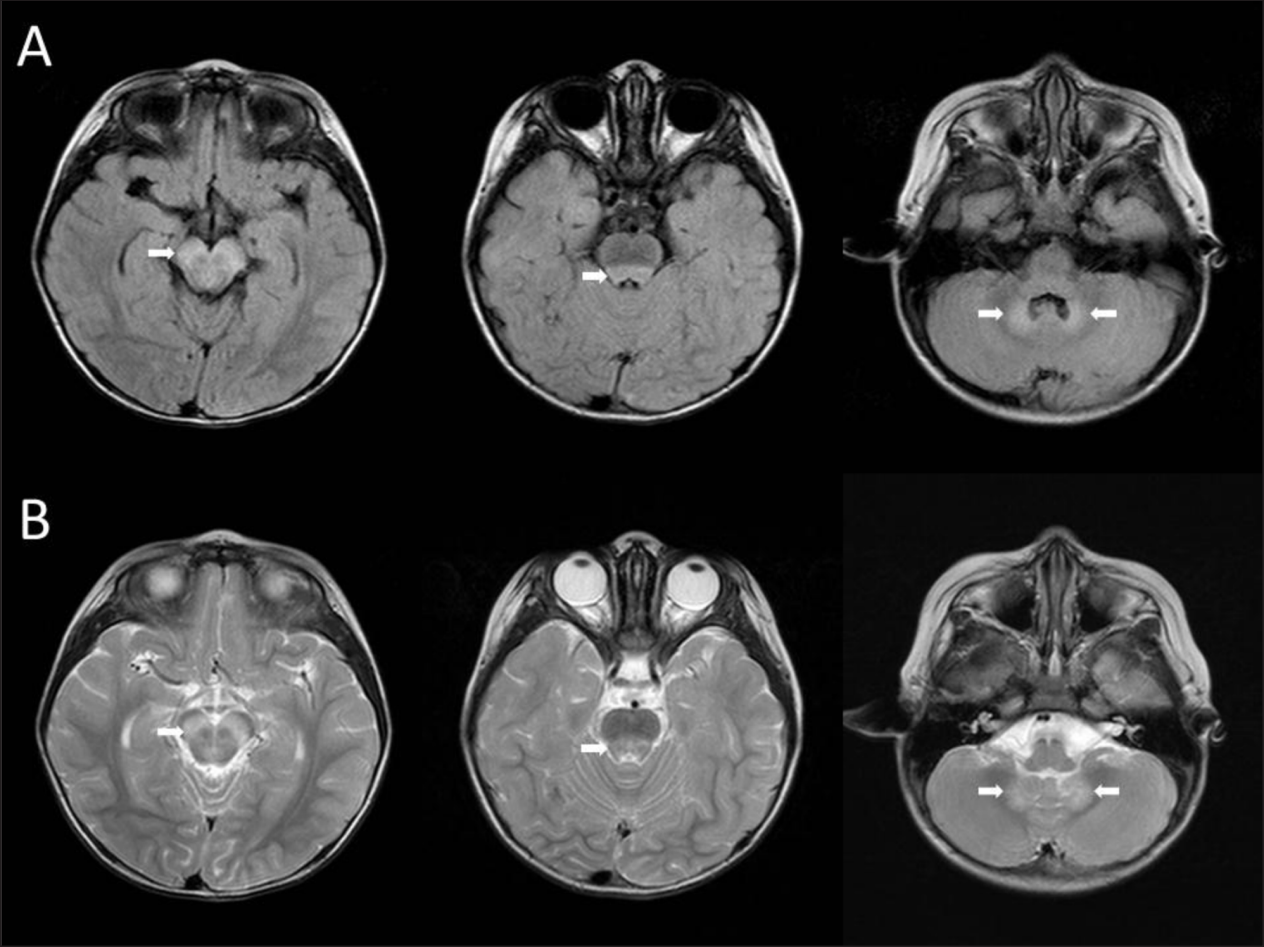
FLAIR **(A)** and T2WI **(B)** demonstrate hyperintense lesions in the midbrain, dorsal pons, and cerebellar dentate nuclei (white arrows).

A two-year-old girl had a fever up to 40°C for six days before admission. She had no cough, rhinorrhea, vomiting, diarrhea, or abdominal pain. She had previously been treated with amoxicillin and cefixmycin prescribed by a local medical doctor for five days, but a low-grade fever persisted. On admission, the results of the hematological and biochemical studies, urinalysis, and adenovirus rapid test were normal. Two days after admission, conscious disturbance was observed. Analysis of cerebrospinal fluid (CSF) revealed white blood cells of 144/μl (lymphocytes 99%, neutrophils 1%), but protein and glucose were within normal limits. Brain MRI revealed hyperintense lesions in the midbrain, dorsal pons, and cerebellar dentate nuclei on fluid-attenuation inversion recovery (FLAIR) and T2-weighted images (T2WI) (Figure 1). These lesions did not demonstrate enhancement after administration of contrast medium. Based on the MRI findings, the initial diagnosis was enterovirus 71 (EV71) encephalitis. Intravenous immunoglobulin was administered immediately after the MRI. However, anti-EV71, anti-mycoplasma, anti-herpes simplex virus (HSV), and anti-Epstein Barr virus immunoglobulin M and immunoglobulin G antibodies were all absent in the serum. EV71 RNA and HSV DNA were not detected in the CSF by polymerase chain reaction (PCR). None of the viruses was isolated in the throat and rectal swabs, CSF, or serum. There was no bacteria growth in blood and CSF cultures. Finally, CVB3 was detected in the CSF by reverse transcription-PCR. The girl recovered gradually and uneventfully, and was discharged without neurological sequelae.

## Discussion

To the best of our knowledge, this is the first report demonstrating the MRI appearance of CVB3 encephalitis. To date, there have been four cases in three reports describing the MRI appearances of pediatric coxsackievirus encephalitis in the English-language literature [1,2,3]. The patient age, serotypes of coxsackieviruses, and MRI findings of the previously reported cases and the current case are summarized in Table 1.

**Table 1:** Clinical characteristics, virological data, and MRI findings of the five patients with coxsackievirus encephalitis in the current case and the literature.

Patient no.	Sex	GA (weeks)	Age at onset of illness (days/PMA in weeks)	MRI Findings	Serotype	Reference

1	M	35	3/35	Periventricular white matter	CVB1	[3]
2	F	36	3/36	Periventricular white matter	CVB1	[3]
3	F	Term	20/NA	Dorsal pontomedullary junction, right basal ganglion	CVB1	[1]
4	F	37	12/39	Internal capsule, corpus callosum, cerebral deep and subcortical white matter	CVB2	[2]
5	F	NA	2 years old	Midbrain, dorsal pons, and cerebellar dentate nuclei	CVB3	Current case

GA: gestational age; NA: not available; PMA: postmenstrual age.

The brain MRI in our case revealed abnormal signal intensity in the midbrain, dorsal pons, and cerebellar dentate nuclei. This MRI manifestation is strikingly similar to those of EV71 encephalitis reported by Shen et al. [4] and Huang et al. [5]. In both reports, the lesions were most commonly located in the posterior aspect of the pons, followed by the medulla oblongata, midbrain, and cerebellar dentate nuclei; this pattern of involvement was consistent in their cases. A similar distribution of lesions can be observed in non-EV71 enteroviral encephalitis induced by poliovirus [6], echovirus 7 [7], and CVB1 [1]. Therefore, brainstem involvement is thought to be characteristic of enteroviral encephalitis.

However, in three cases of coxsackievirus encephalitis (cases 1, 2, and 4; Table 1), MRI reveals cerebral white matter lesions without brainstem involvement. This diversity in MRI findings is likely unrelated to virus serotypes because the MRI results in cases 1 and 2 are different from that of case 3, despite the same serotype (Table 1). Extensive cerebral white matter damage was also encountered in EV71 encephalitis [8]. Verboon-Maciolek et al. [9] and Wu et al. [3] reported cerebral white matter damage resulting from non-EV71 enteroviral encephalitis in neonates. In both reports, the gestational age ranged from 28 to 40 weeks, and the postmenstrual age at the onset of illness was under 41 weeks. In contrast, the patient age ranged from two months to seven years (mean age 25 months) in Shen’s report [4] and 3 months to 8.2 years (mean age 2.5 years) in Huang’s report [5]. According to the above reports, patients with brainstem involvement were older compared to those with cerebral white matter lesions. This observation is significant in image interpretation and suggests that age and brain maturation may play an important role in the pathogenesis and MRI manifestations of enteroviral encephalitis.

## Conclusion

Based on our review, the MRI manifestations of pediatric coxsackievirus encephalitis are age-dependent and similar to those of enteroviral encephalitis caused by other serotypes. Two patterns of involvement are recognized. In neonates, the lesions are distributed bilaterally in the cerebral white matter, without brainstem involvement. Characteristic brainstem involvement, most commonly in the posterior pons, are observed in older children. Although a definitive diagnosis of coxsackievirus encephalitis needs virus identification, familiarity with MRI features is helpful in guiding the management of these patients.
